# Recovery after domestic, family and sexual violence: impact of a holistic clinical support program on victim-survivors

**DOI:** 10.3389/fpsyg.2026.1742876

**Published:** 2026-01-29

**Authors:** Mia Davies, Lata Satyen, John W. Toumbourou

**Affiliations:** School of Psychology, Deakin University, Burwood, VIC, Australia

**Keywords:** clinical interventions, domestic family and sexual violence, domestic violence, holistic interventions, recovery and healing, sexual violence, trauma recovery

## Abstract

**Introduction:**

Recovery frameworks are crucial to mitigate harm and promote positive outcomes for victim-survivors of domestic, family, and sexual violence (DFSV). Despite this, mental health service system responses are inadequate and there is a lack of specialized support programs to promote recovery and healing.

**Methods:**

This study investigated the impact of a novel, holistic, trauma-and-violence-informed recovery framework (the s-CAPE program) on victim-survivors’ recovery from trauma following DFSV, conducted at a gender-specific, trauma-focused clinical setting in New South Wales, Australia. Qualitative interviews were conducted with 10 victim-survivors who had undertaken the s-CAPE program. Thematic analysis was used to derive themes capturing participants’ experiences and perspectives.

**Results:**

Analysis yielded seven themes exploring participants’ recovery journey, their experiences at the service, its impact on their functioning, and preferences for care. Participants largely considered the s-CAPE program beneficial for their recovery and experienced benefits across a range of mental and physical health domains. Recommendations for service improvement were identified.

**Discussion:**

There is a crucial need for more gender- specific, trauma-focused mental health services utilizing holistic and trauma-and- violence-informed approaches to recovery support. These programs need to be accessible and available for broader populations, requiring increased investment, policy change, and collaboration at the systemic level.

## Introduction

While it is widely known that experiencing domestic, family, and sexual violence (DFSV) has long-term, detrimental impacts on health and functioning, and that DFSV pervasively affects one in three women worldwide (World Health Organization [WHO], 2024), there is limited focus on promoting recovery amongst victim-survivors in order to mitigate these effects and promote more positive outcomes. Domestic and family violence involves violent, controlling and/or threatening behaviors, either physical, sexual, psychological, emotional, spiritual, technological, or financial, perpetrated by a current or former intimate partner or other family member (United Nations, 2020), while sexual violence refers to “any sexual act, attempt to obtain a sexual act, or other act directed against a person’s sexuality using coercion, by any person regardless of their relationship to the victim, in any setting” (World Health Organization, 2024). These forms of violence tend to co-occur (McFarlane et al., 2005) and disproportionately affect women (World Health Organization, 2024): in Australia, 1 in 5 women have experienced sexual violence since the age of 15, compared to 1 in 16 men (Australian Institute of Health and Welfare, 2024); globally, 55% of all female homicides are committed by intimate partners or family members, compared to 12% of male homicides (United Nations Women, 2024). Despite these statistics, service frameworks aimed at facilitating recovery are minimal, and supports designed specifically for women affected by DFSV are necessary in order to facilitate wellbeing and reduce harm (Williams et al., 2024).

Recovery frameworks need to support victim-survivors of DFSV to rebuild their lives after trauma, heal the various areas of health, functioning, and wellbeing that have been negatively impacted by these experiences, and reconnect with themselves, others, and the world. Recovery is difficult to define as it is generally recognized to be complex, multidimensional, and non-linear (Carman et al., 2023; D’Amore et al., 2021; Flasch, 2020). It has been argued that during recovery, victim-survivors “reconnect the fragments of the self” (Farrell, 1996) and “move forward… to achieve positive, satisfying lives and relationships, as well as optimal functioning in various areas of their lives” (Flasch et al., 2017, p. 3375). Importantly, recovery extends beyond a reduction of symptoms towards growth, transformation, and “thriving” across life domains (Sinko and Saint-Arnault, 2020; Sinko et al., 2024). This notion of thriving relates to wellbeing, or “a positive state…[which] encompasses quality of life and the ability… to contribute to the world in accordance with a sense of meaning and purpose” (World Health Organization, 2021, p. 10). Thus, reference to wellbeing throughout this paper considers this concept in relation to the thriving aspect of recovery, and as a function of the recovery process. Recovery is unique to each victim-survivor and generally involves changes in a range of areas including trauma processing, emotion regulation, identity, freedom, safety, acceptance and forgiveness of self, developing positive, meaningful relationships, and finding enjoyment and purpose in life (Carman et al., 2023; Flasch, 2020; Sinko et al., 2024). Autonomy, freedom, and independence, as well as self-worth and self-compassion have been identified as integral to recovery and healing (Flasch, 2020; Sinko and Saint-Arnault, 2020). Thus, recovery is complex, and further efforts are required into developing effective ways to promote healing across these domains.

Domestic, family, and sexual violence is known to be a highly traumatic experience, and victim-survivors are at greater risk of experiencing post-traumatic stress disorder (PTSD) as well as an array of negative physical and mental health outcomes, such as increased depression, anxiety, and suicidality (Coker et al., 2002; Devries et al., 2013; Dillon et al., 2013). Rates of PTSD amongst victim-survivors of DFSV range from 30 to 70%, which is much higher than that of the general population (12%) (Nathanson et al., 2012; Pico-Alfonso et al., 2006), and they are more likely to be diagnosed with Complex-PTSD than PTSD (Fernández-Fillol et al., 2021). The trauma symptoms resulting from DFSV are unique and complex – including alterations in affect regulation, self-perception, and perceptions of the perpetrator (Herman, 2004, p. 370), impaired sense of self, identity, and self-esteem (Anderson et al., 2012; Carman et al., 2023), increased feelings of worthlessness, shame, and blaming themselves for the trauma (Basile and Smith, 2011; Cravens et al., 2015; Pemberton and Loeb, 2020), and impaired self-efficacy and decision-making (Sullivan et al., 2018; Williamson, 2010). DFSV is additionally associated with a range of negative outcomes to victim-survivors’ social, economic, and occupational functioning, including social isolation, loss of employment, housing insecurity and reduced life satisfaction (Baker et al., 2010; Bo and Yating, 2023). Thus, effective responses and supports are of crucial importance to mitigate the far-reaching, detrimental impacts of DFSV and facilitate victim-survivor recovery.

Due to the nature of DFSV, victim-survivors risk being retraumatized when accessing mental health and healthcare services (Duckworth and Follette, 2012; Gordon et al., 2025) and thus require unique service approaches. Trauma-informed approaches to service delivery aim to reduce the risk of retraumatization and promote recovery by embedding sensitivity to the effects of trauma across all aspects of service provision (Isobel et al., 2021a; Substance Abuse and Mental Health Services Administration, 2017), incorporating the key principles of collaboration, empowerment, trustworthiness, safety, and cultural sensitivity (Substance Abuse and Mental Health Services Administration, 2017). Trauma-informed approaches have been associated with increased engagement with mental health services, and improved physical and psychological health (Chu et al., 2024; Gilbert et al., 2012; Liu et al., 2024). The contextual and structural factors implicated in violence perpetration and victimization, including social, political, cultural and historical considerations, are essential to understanding the broader sociopolitical and intersectional contexts within which violence takes place (Crenshaw, 1991; Wathen and Mantler, 2022). Trauma-and-violence-informed care (TVIC) incorporates an awareness of these considerations across all services that support victim-survivors of DFSV (Wathen and Mantler, 2022), involving safety, survivor-centered care, non-judgmental, respectful support extending beyond safety, and providing holistic and culturally safe, inclusive services (Davies et al., 2025). Due to the widespread impacts of experiencing DFSV, a holistic approach to support is necessary to comprehensively support victim-survivor recovery across each affected area of health, wellbeing, and functioning. Holistic support has been shown to positively impact the recovery of victim-survivors of sexual violence (Hegarty et al., 2017; Paphitis et al., 2022), facilitate accessibility to services and promote more positive health outcomes for victim-survivors (Mantler and Wolfe, 2018). The World Health Organization recommends a holistic service approach due to the increased accessibility and reduced need for victim-survivors to retell their stories (World Health Organization, 2013).

Holistic trauma-and-violence-informed approaches to care have not been consistently applied in the context of DFSV, and services are not adequately meeting the need of victim-survivors of DFSV, and often perpetuate harm or retraumatization. In research across mostly high-income countries, victim-survivors report experiences of denial, dismissal, invalidation, discrimination, blame, misdiagnosis, and stigmatization when seeking support from various services (Kennedy et al., 2023; Murvartian et al., 2023; Ravi et al., 2022; Taylor, 2020). The power imbalances inherent in mental health services can recreate circumstances reflective of abusive relationships (Oram et al., 2022), particularly as service users have reported practitioners engaging in coercive and controlling practices (Trevillion et al., 2014). For victim-survivors from diverse or marginalized gender, sexual, cultural, and socioeconomic groups, inadequate accessibility, a lack of culturally relevant services, and discriminatory practices are common (Green et al., 2024; Kennedy et al., 2023). A lack of understanding and awareness of sexual trauma among some mental health practitioners often result in feelings of invalidation, distress, retraumatization, and distrust of services for sexual victim-survivors (Gordon et al., 2025; Isaac et al., 2023). Furthermore, service systems are typically overly complex and difficult to navigate (Isaac et al., 2023; Milaney et al., 2019). Evidently, existing service systems that victim-survivors engage with are inadequate at facilitating victim-survivor recovery and healing, highlighting the need for consistent, effective frameworks to support recovery and further research into the efficacy of approaches.

Despite these challenges, formal supports are vital in supporting recovery and healing in the aftermath of DFSV (Green et al., 2024). Research has indicated that engagement with formal supports results in better outcomes for victim-survivors and serves as a protective factor from further harm (Mughal and Saint-Arnault, 2024; Sinko et al., 2024). Victim-survivors can experience an increased sense of self-efficacy and empowerment, improvements in coping abilities and safety, and reduced feelings of guilt and isolation as a result of service engagement (Allen and Wozniak, 2011; Bennett et al., 2004). Interventions supporting women affected by DFSV tend to be shelter-, community-, or advocacy-based (Allen and Wozniak, 2011). However, clinical settings play an important role in facilitating improvements in mental health. For example, formal psychological support has been associated with reductions in PTSD, anxiety, and depression symptoms (Hameed et al., 2020; Trabold et al., 2020; Warshaw et al., 2013). Despite this, little is known about the impact of clinical supports across the broader domains of recovery, health and functioning, and there remains a dearth of holistic, trauma-and-violence-informed clinical recovery frameworks (Williams et al., 2024).

For women or individuals assigned female at birth (AFAB) who have experienced trauma, gender-specific facilities are particularly important. This is because women are more likely to have experienced interpersonal violence and sexual trauma, typically perpetrated by a man (Guina et al., 2019; World Health Organization, 2024), and their PTSD symptoms differ from that of men (Olff, 2017). Thus, gender-specific in-patient programs are necessary to create a safe, effective space for recovery and healing and to avoid further retraumatization.

In the Australian context, the mental health system faces systemic challenges that limit accessibility and availability of services. The public funding model is limited and rigid, particularly for supporting those affected by complex trauma (Salter et al., 2020), and many individuals delay receiving mental health care due to high costs (National Mental Health Commission, 2024). The system is inequitable, with research demonstrating that those most in need of mental health support have the least access (Dawadi et al., 2024). Mental health services are underfunded and experiencing systemic shortages of essential mental health staff (National Mental Health Commission, 2024), as well as a lack of trauma-informed practitioners (Salter et al., 2020). Australian governments have urged that trauma-informed approaches be applied across services including mental health, disability and child services (Department of Social Services, 2023; Department of Families, Fairness and Housing, 2023; NSW Mental Health Commission, 2014). Despite this, the implementation of trauma-informed practices in Australia has been found to be generally inadequate, lacking consistency, cohesion, and guidance (Quadara and Hunter, 2016). Australian research suggests that traumatizing practices continue to be experienced in Australian mental health settings and practitioners experience difficulty translating trauma-informed principles into practice (Isaac et al., 2023; Isobel et al., 2021b; Isobel et al., 2021c). Further research into trauma-informed mental health services is thus essential.

The current study provides the first evaluation of the experiences of victim-survivors of DFSV who have undertaken a holistic, trauma-and-violence-informed program, conducted in a women’s only, trauma-focused clinical setting. It was expected that this study would provide valuable insight into the efficacy of such frameworks in supporting women’s recovery after DFSV and provide an understanding of their utility in the broader health service systems. This study therefore aimed to explore the experiences of victim-survivors who have undertaken the program, their recovery journey and previous experiences with service systems, to develop an in-depth understanding of the impact of the holistic, trauma-and-violence-informed framework on their recovery from trauma, ascertaining the utility and helpfulness of different aspects of the program as well as victim-survivor preferences for care.

## Methods

### Design

This study utilized a qualitative approach to explore the experienced impact of a recovery intervention through participants’ own words in a clinical setting for victim-survivors of DFSV. It formed part of a broader mixed-methods project evaluating this program. The project received ethics approval from the Human Research Ethics Committee (2023-369) of the authors’ institution and funding for the study was provided through a research scholarship program at the institution. This inquiry was conducted using semi-structured interviews and data were analyzed using inductive thematic analysis. Qualitative research allows for capturing, understanding, and describing of complex phenomena (Sofaer, 1999) through this approach, researchers are able to “[understand] the meaning a phenomenon has for those involved” (Merriam and Tisdell, 2016). Thus, qualitative data can provide a comprehensive understanding of participants’ lived experiences engaging with the program, and in-depth insight into their experiences of its impact and efficacy that cannot be captured using quantitative data.

The researchers involved in this project have extensive experience in the field of DFSV research, policy and practice and have previously worked on projects investigating recovery frameworks in the DFSV sector. This prior experience may be reflected in their interpretations of the current findings. The first author has researched TVIC frameworks for supporting DFSV recovery; therefore, her understanding of this theory may have influenced her interpretation of results. Clinical work has strengthened her understanding of the lived experience of individuals affected by mental health difficulties, although may have influenced interpretation of the data. Identifying as a woman from a Western background circumscribes understanding of diverse experiences. The second author has worked with women affected by DFSV for over 20 years as a practitioner, advising on policy and practice, and conducting research across diverse communities; thus her experience strengthens the understanding of women’s experiences in this study. The last author, as a male professor in health psychology has worked with and supported organizations and students to design, implement and evaluate services. He recognizes he has only indirect understanding of the experience of women affected by DFSV. A reflexivity journal was kept by the first author across the data collection and analysis and the researchers met throughout the research process, reflecting upon their interpretations of the data.

### Setting

The Ramsay Clinic Thirroul (RCT) in Thirroul, New South Wales, Australia, is Australia’s first women’s only, trauma-focused treatment facility, offering in-patient and out-patient programs aimed at supporting women’s recovery from trauma, typically complex interpersonal trauma. The clinic began operating in 2022 and is privately funded by Ramsay Health. Developed in response to service delivery problems within the Australian context and the need for holistic approaches to recovery in clinical settings, the s-CAPE program (Williams et al., 2024) conducted at the RCT offers a holistic, trauma-and-violence-informed service for recovery from trauma. S-CAPE is delivered by trauma-and-violence-informed specialist mental health practitioners and focuses on safety, stabilization, emotion regulation, skill-building, community and social support, attachment, processing, physical health and well-being, developing creativity and enjoyment, and empowerment. The program comprises of three phases, with each constituting a three-week in-patient stay at the RCT, offering bridging day programs between stays. Phase One focuses on establishing safety and stabilization; Phase Two involves building emotion regulation, beginning to process trauma, and the recognition of trauma, while engaging in a range of therapeutic programs; Phase Three focuses on supporting the recovery and healing of women through an extension of a scheduled program of clinical and non-clinical support programs. S-CAPE involves group therapy sessions as well as one-on-one psychological and psychiatric support. Additionally, an in-patient stay at the RCT includes holistic supports such as nutritional therapy, trauma-informed physical exercise support, pet therapy, art therapy, and well-being activities in nature. At the time of data collection, around 1,500 individuals had received support at the Ramsay Clinic Thirroul.

### Participants

Participants were individuals who were either current in-patients at the RCT or were receiving support through out-patient services but had previously completed an in-patient stay. Participants in the in-patient program at the time of interview were included only if they had been in the program for at least 2 weeks or had had a previous admission of at least 2 weeks. To be admitted to the RCT, individuals needed to identify as a woman or assigned female at birth, were aged 18 years and above, had experienced significant trauma and a diagnosis of PTSD or CPTSD. Significant trauma in this context refers to traumatic experiences of a severe, long-term, or repetitive nature, involving severe psychological or physical injury of an interpersonal nature, sexual harm, and/or disempowerment. To avoid distress, participants were not asked about the specific traumas they had experienced, however, staff at the clinic confirmed that all individuals that were receiving support from the RCT at the time had experienced interpersonal trauma, either domestic and family violence or sexual violence, often repeated, and multiple types of traumas. Staff at the RCT provided recruitment information about the project during the period of October and November 2024, and directed participants to contact a member of the research team if they were interested in participating. Participants were informed that participation was entirely voluntary, and they were able to withdraw their consent to participate at any time. Participants were also reassured that their participation or withdrawal of consent from the study would have no impact on them receiving any services from the RCT. Those interested in participating returned a signed consent form. Research indicates that between 9 and 17 qualitative interviews are required before data saturation is reached (Hennink and Kaiser, 2022); thus, this sample size was the aim for recruitment.

### Procedure

Participant interviews took place in November 2024 either in person at the RCT or online via Zoom, depending on the participant’s preference and availability. Before commencing the interview, participants were reminded that they were able to take a break, terminate the interview, or receive support from trained, mental health professionals at any time during or after the interview. Interviews followed a semi-structured format. The questions were derived from an interview protocol developed by the research team to capture participants’ experiences receiving care from the RCT. Rather than using an existing questionnaire, an interview protocol was developed to encourage exploration of participant experiences in a manner that reflects the specific context and addresses the research questions. Semi-structured protocols allow for flexibility and versatility by avoiding limiting the responses to a preconceived set of ideas (Magaldi and Berler, 2020). Protocol questions included “What changes, if any, have you noticed in your mental health since receiving support from the Ramsay Clinic Thirroul?”; “How do you think services could best meet your needs for recovery?”; “Are there any parts of the Ramsay Clinic Thirroul service that you particularly liked or that you found particularly helpful? If so, what were they? If not, why do you think that is?” With the participant’s consent, the interviews were audio-recorded. The interviews ranged between 30 min to 1 h. In line with recommendations from the Australian National Research Agenda to End Violence against Women and Children 2023–2028 (Lloyd et al., 2023) and the Victim-Survivor Advisory Council guidelines (State Government of Victoria, 2023), participants were reimbursed following the interview with an AU$100 voucher to adequately compensate their valued time and expertise, and to balance the risks of participating in research as people with lived experience. They were also provided with a list of DFSV support resources.

### Analysis

Interviews were transcribed verbatim by the researcher who interviewed participants. MAXQDA qualitative data analysis software was used for analyses (VERBI Software, 2024). Data analysis followed a reflexive thematic analysis approach, as outlined by Braun and Clarke (2006). Reflexive qualitative analyses rely on an active role of the researcher in interpreting and engaging with the data, rather than a pre-established codebook, theory, or hypothesis of the results (Braun and Clarke, 2021). Thematic analysis was described by Clarke and Braun (2017) as “a method for identifying, analyzing, and interpreting patterns of meaning (themes) within qualitative data” (p. 297). An inductive approach was used in the current analysis, which is a form of reflexive thematic analysis that is ‘grounded’ in the data (Braun and Clarke, 2021), whereby researchers develop themes through interacting with the data itself, rather than reliance on pre-existing theory. This process involved the researcher first becoming familiar with the data by reading through the transcripts. The researcher then generated codes from the dataset capturing the meaning of quotations and essential concepts across transcripts. Two researchers then organized the codes into initial themes based off code groupings, developing an initial thematic map. The themes were then reviewed by the research team and adapted to develop a final thematic map. The themes were then named and defined in a meaningful way in relation to the research questions, with sub-themes identified.

## Results

**Figure 1:**
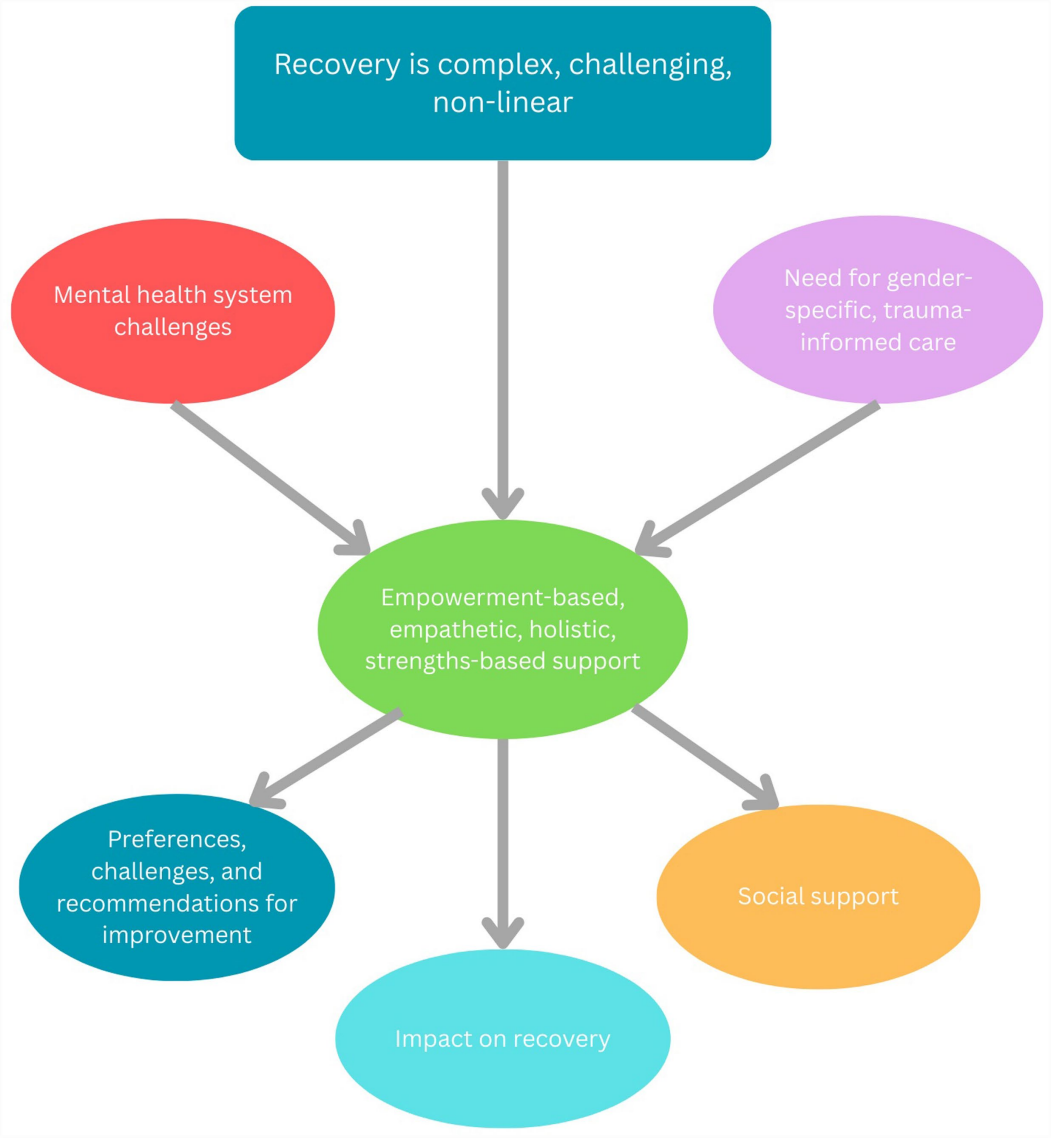
Thematic structure and conceptual relationships.

A total of 10 interviews were conducted. Demographic characteristics of participants are presented in Table 1. All participants had engaged with previous mental health supports including psychologists, psychiatrics, mental health in-patient admissions, counsellors, sexual assault counsellors, mental health day-programs, and psilocybin-assisted psychotherapy. All participants had spent at least 2 weeks of an in-patient stay at the RCT; half had completed Phase Two of the program, and half had received out-patient psychiatry or psychology support. The length of time for which participants had been engaged with the clinic ranged from between 2 weeks to 2 years. No participants withdrew throughout the research process. Two of the 10 interviews were conducted online; these participants were not admitted to the in-patient program at the time of data collection and were receiving out-patient support. An additional two participants were receiving outpatient support at the time. Thematic analysis resulted in the development of seven themes, each with sub-themes, describing victim-survivors’ experiences receiving care, both at the RCT and in the broader mental health system, their experiences with recovery, and the impact of the holistic, trauma-informed clinical program on their recovery in the aftermath of DFSV. Figure 1 displays the themes and maps the relationships between them, demonstrating how the need for such a program arose and its outcome. As indicated by Figure 1, the complex nature of recovery, challenges within the mental health system, and an expressed need for gender-specific, trauma-informed care has given rise to the development of a program characterized by empowerment-based, empathetic, holistic, and strengths-based support to meet these needs. The findings thus reveal the impacts of such a program on recovery including the primacy of social support, and the resulting recommendations to further improve mental health and healthcare services.

**Table 1:** Demographic characteristics of the sample.

Characteristic	Result
Age (years), mean	37.7
Range	23–60
Gender identity	1 intersex 9 women
Cultural identity	8 Anglo-Australian 1 British 1 South-East Asian
Time engaged with RCT, mean	~7 months
Range	2 weeks–2 years
Type of support at RCT, proportion	100% 2 weeks of Phase One 60% started Phase Two 50% completed Phase Two 30% day-program 50% out-patient support

### Theme 1: mental health system challenges

The participants identified mental health system challenges and barriers. Many described negative and harmful previous experiences with supports, an over-reliance on medication, and a lack of trauma awareness. Systemic barriers within the mental health system were identified, including challenges with affordability, accessibility, and availability, noting a lack of women’s-only, trauma-specific supports.

#### 1a: negative, unhelpful, and often harmful

Participants had previously accessed a range of mental health supports, including in-patient stays, psychologists, psychiatrists, and counsellors. Many mental health system experiences were described as either negative, unhelpful, or causing further harm, including being dismissed, invalidated, and criticized by mental health professionals, as well as feeling disempowered during invasive room and body searches. Some in-patient experiences were re-traumatizing, described as “horrible”; “terrifying”; “traumatizing and confronting.”

There was a lack of understanding of trauma, and trauma was often overlooked despite being the source of difficulty. Mental health services reportedly over-rely on medication to manage trauma symptoms, with distress often responded to with increased types or doses of medication. Many participants’ functioning was significantly affected by high doses of medication, and they felt disempowered and frustrated by these experiences:

I feel angry at the doctor who prescribed me all this medication that I’ve been taking for 9 years that I do not even need to be on. And it’s affected my job. It’s affected my life. It’s had a knock-on effect of every aspect of my life.

Despite this, some mental health supports were noted to be helpful, particularly trauma-informed and long-term psychologists or counsellors, and the relationships formed with these practitioners were identified as facilitators of successful support.

#### 1b: systemic barriers and challenges

Barriers and challenges to receiving effective mental health support at the system level included issues with accessibility and affordability. Wait times for mental health practitioners were very high and practitioner availability was limited. Participants reported that mental health services and insurance premiums were very costly, particularly for those with significant mental health concerns. Participants noted that traumatized women are more likely to be those with limited access to resources, thus raising further concerns for accessibility. Participants expressed concerns that women’s only, trauma-specific hospitals were extremely limited, with RCT being the only one in Australia and therefore unable to meet Australia-wide demand. The service was also only available to those who had private health insurance, live locally or had the means to travel. Participants expressed a desire for the development of public services like the RCT: “I love the facility, and I love the place. And I do wish it was more available to a greater range of women, not just women who could afford it.” Furthermore, the current service system was described as “very fragmented,” and service integration was identified as essential to effective and comprehensive responses.

### Theme 2: the need for gender-specific, trauma-informed care

The interviews highlighted the need for gender-specific, trauma-informed support in mental health services, with participants strongly valuing TIC. Women’s-only, trauma-specific support was identified as crucial for safety and healing in in-patient settings for women affected by trauma.

#### 2a: trauma-informed care in the mental health system

Trauma-informed care (TIC) was described as being strengths-based and non-judgmental, involving kindness, compassion, empathy and attunement, upholding victim-survivors’ lived expertise, seeing them for who they are, and not imposing one’s own views. Its relational nature was emphasized in the following:

Trauma-informed practice is really about relationships and it’s about having the safety and the ability to be seen and to be heard and the connection that you kind of have and even though it’s still within that clinical construct, it’s the people that mattered.

Participants identified the importance of trauma-informed support, and this approach was considered the most valuable and helpful attribute of a mental health practitioner. Participants largely described the mental health system as inadequately trauma-informed, and a lack of TIC was considered to lead to misdiagnosis and inadequate treatment. Participants advocated for more trauma-informed mental health services. Service integration was argued to be necessary for a trauma-informed system: “until we start working collaboratively and… as more of a collective, then we are not going to see change.”

#### 2b: benefit of women’s-only, trauma-specific support

When asked why they chose the clinic, many participants identified that it was because it was a women’s-only clinic and/or provided trauma-specific support as the primary reason, and this was considered one of the most helpful aspects of the program. It was noted that ‘trauma’ presents differently across genders, creating difficulty in combined settings, including women’s mental health concerns being overlooked as they were more likely to be internalized. Gender-specific care was considered particularly important for safety. Many participants reported feeling safer in a women’s-only environment, particularly as the sources of many women’s trauma were experiences with men, which also made them fearful of being subjected to sexual assault or aggression. The importance of a trauma-specific environment was also emphasized as being in in-patient settings alongside consumers with other acute mental health concerns could lead to fear and worsening trauma symptoms. The clinic was therefore identified as a safe space, allowing victim-survivors a respite in which to work on healing and recovery – a setting that was not available elsewhere.

### Theme 3: recovery from DFSV is complex, challenging, and non-linear

Participants highlighted that recovery is complex and non-linear, challenging, and ongoing journey. The nature of recovery and the pervasive negative impacts of trauma on all areas of health and functioning meant that trauma-focused therapy was recognized as difficult and taxing, involving a process of slow and gradual change.

#### 3a: conceptualizations of recovery

When asked to identify what recovery meant to them, participants provided rich, expansive, and descriptive conceptualizations which conveyed the challenging, complex, and multidimensional nature of recovery. Recovery was described as an ongoing and lifelong process, a “journey,” and a daily struggle. Participants emphasized that recovery did not have an end point as one may not “fully recover”; however, they indicated that people will continue to grow and move in a general direction of progress and healing:

I do not think it’s, like, ever going to end as such… I do not think there’s going to be like an end date that I’ll be like, hey, I’m done. I just think, it’s just will not have to be so intense forever.

One participant poetically described it as “an unpredictable destination… that leads to anywhere but here.”

The recovery process was described as non-linear and “sometimes one step forward and two steps back,” involving “ups and downs” and a mix of both “wins and griefs.” Trauma was described as “leaving scars” that an individual learnt to live with over time, eventually reducing their pain. Recovery was also characterized as becoming “unstuck,” with one participant applying a metaphor of preparing instant noodles:

You, like, start doing the work, and it’s like pouring boiling water over the noodles and it’s hot and it’s uncomfortable. But like, everything starts moving, and… it’s so hot and uncomfortable. But… everything that you have started to believe, starts to shift and it starts to move and… kind of make a little bit more sense.

Another participant expressed the idea that recovery involves learning – learning strategies, new ways of coping, how to be kind to oneself – and gradually and effortfully applying this learning with the aim that it eventually becomes second nature and no longer effortful. Recovery was considered to be multidimensional and supported by different processes, including medication, lifestyle choices, therapy, trying new experiences, managing expectations, connection –with family, friends, pets – purpose, acceptance, grief, hope, and forgiveness, of oneself and of others – “a free fall and stumbling to find the self.”

#### 3b: trauma-focused therapy is challenging

Participants expressed that trauma-focused therapy is challenging and takes time, and this was due to the widespread and pervasive negative impacts of experiencing complex trauma. Trauma affected many parts of participants’ lives and they were often unaware of the extent of its impacts. Participants reported feelings of hopelessness, guilt, shame, suicidal ideation, low mood, anxiety, numbness, inability to connect with emotions, irritability, negative self-cognitions, lack of motivation, physical symptoms (i.e., tension, tremors, and pain), sleep difficulties, disordered eating, social withdrawal and isolation, and difficulty trusting, communicating with others, or asking for help. Trauma often damaged one’s sense of self and impacted functioning across life domains (e.g., relationships and work). Trauma-focused therapy was thus identified as challenging and taxing as it involved “rewiring the brain” and overcoming longstanding, pervasive difficulties:

Trauma is kind of the way that your brain is shaped. And so it’s not gonna be a quick fix from, you know, being in a three-week program or 3 weeks times two, that’s going to kind of magically change I guess the way that you think or the way you think of the world or the way that you think of yourself in the world.

Participants felt frustration when engaging in many practices to support their recovery without seeing substantial changes: “That’s where I got to I was like… I’m doing all the things I was told to, I should feel better by now….that led to the conversation around decreasing the expectation [on myself].” It was acknowledged that this work takes time and involves gradual changes over time: “It goes through peaks and troughs but… the trajectory is getting better.”

### Theme 4: empowerment-based, empathetic, holistic and strengths-based support

Models of care that are holistic, empathetic, respectful, empowering, and emphasize safety were favored by participants and identified as necessary when providing support to those affected by DFSV. Participants largely indicated that these principles aligned with the care they were provided at the clinic.

#### 4a: respect, autonomy, and choice

The approach to care characterized by respect, autonomy, and choice was favored, considered necessary in trauma support to avoid retraumatization and foster empowerment. This involved recognizing and adapting to individual needs, such as allowing flexibility to say no and choose what works for them: “the groups aren’t forced upon you, I’m able to have control over it and do my own thing if I would rather that, given flexibility to be able to do the stuff that I know helps me.” Respect and autonomy were considered fundamentally important for those affected by DFSV, upheld by practices including being asked permission to enter rooms, being allowed to have personal possessions during their time at the clinic, no invasive searches, and being responded to with gentleness and flexibility when victim-survivors were in distress. One participant described hiding under her desk when distressed and appreciated being allowed to remain there until ready to leave. Participants appreciated being consulted frequently regarding service improvement strategies and provided with many feedback opportunities: “They tried to include the patients as much as possible in any discussions… creating a safe space for patients actually to be able to speak up if there was something they were not happy with.” The importance of balancing power dynamics between practitioner and client was also discussed:

Being partners is key and you know, working to find a balance of power and sharing of responsibility is key and I think that’s the stuff that I think makes the difference for me, and that’s what’s made the difference for me.

#### 4b: empathetic, relational support

Participants emphasized the importance of empathetic support, feeling supported and understood. Trusting relationships with staff were crucial, requiring staff to be caring, warm, compassionate, kind, and respectful – these relationships were considered unique for an in-patient setting:

I feel like the nurses genuinely care here, like they actually know who I am and what my story is and they actually, genuinely are there if you want to check in, like I’ve never had that experience before anywhere. Like normally my experience is go back to your room and they do not want to check in with you.

It was necessary for all staff to utilize this approach, regardless of position or role, as reflected by one participant describing interactions with cleaning staff: “They’re just normal people that they care about you, like they acknowledge that you are here and you are having a rough time … they are willing to listen.” These relationships allowed participants to feel comfortable and empowered to share any difficulties they were having while at the clinic: “If anything happens that’s triggering, I’m able to talk to someone I trust.” Trusting relationships were crucial as a source of ongoing recovery support after leaving the in-patient program: “It’s not necessarily dependent on whether I’m there or whether I’m not. I still have [practitioner’s] support behind me and that, makes me feel like I’m not alone.”

#### 4c: safety

While safety was a challenge for many participants initially, they reported that the clinic was safer compared to other mental health support systems. The focus of Phase One was stabilization and familiarization with the clinic and participants indicated that this supported their safety: “Coming here for Phase One has allowed this to feel like a safe space to feel comfortable in before I come back next time and do the more intensive trauma work.” A sense of safety allowed participants to more easily access and process emotions. They described the clinic as a safe space and respite to focus on themselves, away from the busy demands of everyday life. This allowed participants to be more accepting of needing support, which was healing in itself, as elucidated in the following statement: “It was OK to kind of still be a really strong and influential person and be there to kind of have some time for myself, to kind of focus on my own kind of sense of self and healing” Some participants reported never experiencing safety before and therefore found it challenging to recognize and understand this aspect, particularly emotional safety. Support at the clinic fostered this understanding:

They talked about it all the time, about creating safety. And… in my brain, I was like, what even is that? Because… safety for me has always been a very physical thing… well I’m not about to die there’s not bombs going off outside. So I was just very confused by the whole concept. I never considered like emotional and psychological dangers as dangers… But I think coming to understand emotional safety like all of those things are actually just as significant.

#### 4d: group work

Participants identified the group work as an area they found most beneficial, described as “being in a room around other people who have gone through something as well and are able to understand more so than other people… to be in a safe environment when I can talk to others who understand.” Participants preferred groups that relied on “sitting in amongst the group dynamics” to be therapeutic, rather than on being provided with information, such as groups with a “classroom kind of style.” Closed groups with consistent group members and facilitators were preferred as these allowed participants to feel more comfortable sharing: “the trust thing for me is really hard… it helps that there were a number of ladies there I had crossed paths with and the facilitators were consistent … it was helpful the consistency of who you would have.”

#### 4e: holistic supports

Participants valued the holistic nature of the program, describing a range of activities and therapies offered at the clinic which they found helpful for their recovery, including art therapy, pet therapy, nutritional therapy, trauma-informed exercise including swimming and gym programs, and therapeutic group activities: “It’s a holistic thing… the whole thing is aiming for you to get well, and it’s good.” Participants recognized the importance of physical activity being facilitated in a gentle, trauma-informed and survivor-centered manner and tailored to each individual:

It makes it feel like it does not matter where you are in your fitness level. There are people here who can barely walk, and there are people here who are, like, personal trainers and… [physiotherapist] finds a way to make a plan with you that you can keep doing outside of here.

Pleasant, enjoyable, and calming activities were a source of relaxation and grounding which, as described by one participant: “For some women… gave them an opportunity to not feel like they had to talk, not feel like they had to participate, they are participating in in just their presence and the focus that they had in what they were doing.” These activities also fostered connections between others at the clinic. Participants also discussed the importance of the physical environment as a source of wellbeing: “Being out and seeing the bush and the ocean and everything is so healing.”

### Theme 5: impact of holistic, trauma-informed treatment on recovery

The holistic, trauma-informed program was described by participants as beneficial across a broad range of areas of health and functioning, with most participants considering it helpful for their recovery. Participants reported an increased ability to understand and process their trauma and related emotions, improvements in their mental health and coping skills, enhanced self-compassion, changes to difficult cognitions, improvements in physical health and intrinsic motivation, and reduction in medication usage.

#### 5a: holistic, trauma-informed treatment supports recovery

The majority of participants reported that engagement with the clinic had been beneficial in supporting their recovery from trauma, some describing it as “life changing.” This was experienced even by those who had been unaware that they needed support: “I did not realize that I needed it so much… and then my psychologist said no, you need to go and I’m so pleased that she said that because it really has made a difference.” Participants reported changing behaviors and implementing strategies after leaving the structured program. They described growth as a result of the program: “People are telling me here that I’ve ‘grown so much’ since I arrived. I think I’m seeing parts of myself that needed a safe space to grow.” One participant reported that the program had not been helpful for their recovery and had worsened symptoms, attributed to challenges with the in-patient environment and feeling their experience had been invalidated.

#### 5b: improvement in understanding and processing of trauma symptoms and emotions

A major benefit that participants identified from the treatment was improving their awareness and understanding of the impact of trauma, allowing them to connect to their feelings and to begin processing their traumatic experiences. Many participants had experienced longstanding symptoms affecting their cognitions, emotions, and behaviors, often without awareness of why, and having extensive negative impact on their wellbeing. For some participants developing this understanding of their experience, their traumas, and trauma symptoms was one of the most significant benefits for them:

I think just understanding myself… that level of understanding has helped me a lot in that I can make more sense of things. Because back in the day, like when I was struggling, not being able to make sense of it… that was the hardest thing for me.

Many participants reported being able to connect with and understand their emotions more, after initially being numb or avoidant: “I’ve found it easier to … I guess learn to be more vulnerable or start to learn to like explain emotions or understand emotions.” This connection occurred through the overall experience of feeling supported at the clinic, being in a safe space where emotions felt manageable, and through specific emotion-related work in group therapy.

#### 5c: improvement in mental health and coping skills

Participants reported improvements in their overall mental health and ability to cope with difficult experiences after receiving the treatment. They indicated an improvement in depressive symptoms, including improved mood, a reduction in suicidal ideation, and increased energy, for example:

I had suicidal thoughts for months and months before I came in here. And then I have not had any since I’ve been in. And I was like flooded before I came in here with, like, overwhelming sadness and shame that I could not cope with on the outside every day. And in here I have not had that at all… I have had… moments of… sadness and stuff, but like it has not been like, overwhelming and it’s been… it’s been such a relief.

The treatment fostered an increased sense of coping in participants, and they felt better able to manage day to day life. They reported implementing strategies to manage distress (e.g., journalling, breath-work, taking breaks when needed, and wearing bodily items such as bracelets to minimize self-harm). They experienced improvements in emotional reactivity, an increased ability to pause before responding to difficult experiences, and being “better at just breathing through and processing things than I used to be.” Furthermore, participants reported an increased ability to recognize mental health warning signs and respond accordingly, as characterized by one participant:

The biggest change would just be the awareness piece… I can tell now when I’m like slipping or I know when I need to change or do something. So, I do not fall, I guess, as much as I had previously been like into a slump.

These changes were an important source of hope for participants as they discovered: “there are ways to manage that where you can minimize that or even heal from that. It’s really reassuring.” Conversely, one participant reported a worsening of mental health symptoms following treatment at the clinic, including worsened mood and increased sense of hopelessness.

#### 5d: cognitive restructuring and enhancing self-compassion

Participants reported cognitive changes as a result of the program, particularly changes in cognitions about themselves. While self-compassion was regarded as a challenge, many were able to foster more positive ways of thinking about themselves, particularly through group therapy activities. One participant explained this shift as follows:

I have learned some more self-compassion… why I thought I was just like lazy or I had some sort of moral failure, which is why I could not get over where I was in my life and all my troubles… What I am is, I am having a normal response to a horrible life which a lot of women here are.

Another participant described being able to see themselves from a “point of strength” rather than a “point of weakness.” Developing self-compassion involved reducing the shame, guilt, and self-blame for trauma that is typically experienced after DFSV. This process was described in the following account:

I guess a bit of validation around the situation and the circumstances and it’s not necessarily like me, I did not do this…. it’s not my fault… these aren’t things that I’ve created for myself or whatever; they were ultimately things out of my control.

This was reported as one of the most beneficial changes from the program. Some participants also reported an increase in self-confidence. By contrast, one participant reported worsened thinking about themselves following treatment from the program. Another significant change in cognition involved participants feeling more deserving of receiving support. For example, one participant who experienced debilitating migraines experienced feelings of “not being worthy enough or not deserving enough of support” such that they would never seek help and functioning was severely impacted, reported feeling “more deserving of help” after treatment. Other cognitive changes as a result of the program included challenging unhelpful thinking, reduction in black and white thinking and catastrophizing thoughts, and learning to prioritize one’s self.

#### 5e: impact on dissociative symptoms

A few participants discussed dissociative symptoms with the impact of the program on these symptoms being mixed. While reporting a decrease in dissociative symptoms, participants attributed this to interacting with others who shared similar experiences, causing them to feel less disconnected from reality: “I feel like coming here, like seeing people struggling with the same kinds of things, I’m like ‘oh I’m not an alien’.” Another participant attributed feeling less disconnected from reality to their overall improved health. A worsening of their dissociative symptoms during their in-patient stay was attributed to being in a new and unfamiliar environment where they were less in control of their surroundings than at home. However, they believed this could be a beneficial process: “It’s not necessarily a bad thing, it’s helping me identify that this is something that’s happening, it’s just different to being at home where everything is familiar.”

#### 5f: improvements in physical health

All participants reported improvements in their physical health and wellbeing, which were acknowledged to contribute to improvements in mental health. They reported engaging in more physical activity, including walking, yoga, swimming, and exercising in the gym, improvements in fitness and balance, and a changed personal relationship with exercise. These changes were attributed either to the trauma-informed exercise program at the clinic, or the experiencing of getting out into nature that the program encouraged, and were sustained by those who had left the clinic. While still difficult for some, many participants experienced improvements in their sleep since engaging with the clinic, which led to feeling more energetic and alert. Most participants appreciated the food and nutritional support provided at the clinic, acknowledging the benefits of good nutrition which included increased energy. Participants with restrictive eating disorder symptoms noted an increase in their food intake.

#### 5g: intrinsic motivation to engage in wellbeing

Participants experienced increased intrinsic motivation to engage in meaningful, wellbeing-related activities due to the program. Some described returning to previously enjoyed activities, such as arts and crafts. Others noted a general increase in activity and motivation, physically, socially, and otherwise. Some experienced increased confidence and engagement with life experiences, such as feeling “willing to try things or back myself in doing things that I would not have previously done… very much give it a go kind of mentality now which wasn’t what it was previously.” They described learning to manage expectations of themselves and participate in activities in a more balanced and sustainable way: “I’ve changed the measure of it… it’s not so much about hitting a particular goal… it’s more about just doing something or going outside.” Some participants also discussed having achieved their goals set at the beginning of treatment, which was a source of pride and motivation, and being motivated and able to expose themselves to previously avoided situations while at the clinic.

#### 5h: reducing reliance on medication

One impact of the program was a reduced reliance on medication and reduction in medication usage. As previously discussed, many participants reported that the over-reliance on medication in prior supports had negatively impacted their functioning. While their individual choices were still strongly respected and medication was encouraged when needed, participants were directed away from a reliance on medication for coping and encouraged to utilize other methods:

I’ve even had like situations where… an inpatient and I was … super distressed and I was, like, give me something [reference to medication]. And then I remember one of the nurses [saying] is there another way that… we can manage this before we go to that? Because at the end of the day…that’s what you need to be able to do in life.

Participants were supported to understand the medications they were taking and their effects, to make informed choices about usage, and make changes in a safe and feasible way: “I feel more confident around my medication.” Participants reported feeling more alert, motivated, present, engaged, and connected with their emotions due to changes in medication. For some, this was the most beneficial and consequential impact of the program.

### Theme 6: social support and recovery

The social support and connections developed at the clinic were valued by all participants and identified as highly beneficial to their recovery. Participants also noted changes in the way they relate to others socially, encouraging the development of healthier relationship, leading to improvements in social health.

#### 6a: benefits of social support

Participants discussed the social support and connections developed at the clinic as a fundamental benefit of the program. Connecting with others provided a source of validation, reassurance, empowerment, and relief: “There’s more people around that feel the same way I do …. And I guess it was like, I do not know if the right word is like a weight off, but I just kind of felt like I could breathe.” Many participants had developed valued friendships because of time spent at the clinic and felt that these relationships supported recovery and “growing just from being together.” These connections allowed participants to recognize their own progress as they shared with each other the growth they saw. Most participants experienced increased motivation to socialize outside of the clinic after treatment, which was particularly important as many had withdrawn socially.

#### 6b: supporting healthy social relationships

Participants experienced changes in how they related to others socially, thus supporting healthier social relationships. The program had improved participants’ communication skills, including their ability to express their feelings, which led them to share their experiences with others and seek support more. Reduced reactivity during communication was described as positively impacting relationships. For example, participants reported an increased awareness of the negative impacts of certain problematic relationships. They were also supported to establish appropriate boundaries with others, which involved improving their ability to advocate for themselves. Some participants experienced a changing understanding of relationships, which could be difficult as it involved challenging long-standing processes, as in the following:

It was also trying to… deepen relationships with… those key kind of friends and let them in more… one of the other kind of things that I was challenged with from my like psychologist and psychiatrist which frankly was… just as hard as the setting boundaries thing so… Everything for me… for as long as I can remember, has always been very transactional in terms of relationships, friendships, whatever.

This reportedly strengthened their connections, such as friendships.

### Theme 7: preferences, challenges, and recommendations for improvements

Participants identified challenges engaging with the service and recommendations to improve support, including systemic difficulties such as staffing and communication, and challenges resulting from staff knowledge, other service users, or transitioning out of in-patient support, upholding the importance of continuity of care.

#### 7a: challenges with overall service

Participants reported some difficult experiences and challenges with the overall service. Some aspects of the program were less trauma-informed than others, as discussed by one participant: “There’s parts that are, there’s parts that aren’t. There’s [sic] things that they could improve on and there’s [sic] things that they have really tried to do well.” This included hourly room checks throughout the night which felt invasive, particularly when conducted by unfamiliar staff. The employment of male staff negatively impacted some participants’ feelings of safety, although others acknowledged it was helpful in exposing them to safe males. This tension was described by one participant: “Even being around some of the male nurses has helped a lot. There have been ups and downs. It has been hard having male presence around, some are really lovely.” Participants identified challenges with communication, including from staff to service users, with some feeling inadequately informed during their in-patient stay, and between staff members: “Sometimes you talk to one nurse about something and then you speak to another and they do not know what you are talking about, so communication between them about what’s going on would be helpful.” Some participants reported instances of medication mismanagement, which significantly impacted feelings of trust and safety during their stay. Most difficulties with the program were attributed to “teething problems” as the program was relatively new: “This place feels very new. It feels like they are still like, figuring stuff out. You can tell.”

#### 7b: staff knowledge and its impacts

Difficulties with staff and staff knowledge impacted participant safety and comfortability. Instances of high staff turnover or being understaffed caused casual nursing staff to be outsourced from other hospitals. These were not necessarily trauma-informed, were unfamiliar, sometimes unfamiliar men, impacting feelings of safety and causing distress. Some participants described inconsistent experiences across staff which created challenges, characterized by one participant as:

Really hit and miss and I just got to the point where I knew who I could go to and who I who I wasn’t going to go to… I’d much rather sit it out and wait it out until that person came on that I felt comfortable or felt they were going to… listen.

Experiences of staff engaging in behavior that was not trauma-informed were reported: these included removing items from rooms without consulting service users, miscommunications around permissible activities that resulted in participants feeling “belittled and panicked,” instances where service users felt pressured to engage in activities when highly distressed, feeling invalidated or not listened to, and where they felt boundaries in terms of private information were not respected. The difficulty of shifting practices that were common occurrences in the mental health system was acknowledged, emphasizing the need for consistent and ongoing training.

#### 7c: challenges with other service users

While connections with other service users were highly valued, participants also noted some difficulties around interacting and inhabiting the clinic with others affected by DFSV. Managing different group dynamics was challenging as the group members varied and different personalities, expectations, and approaches produced tensions. While beneficial in many ways, being around others who had experienced trauma could create difficulties. It was confronting for some participants to hear information about traumatic experiences shared outside of group therapy settings. The importance of maintaining boundaries with other consumers was discussed, and for some participants it was emotionally taxing to form connections with others during the inpatient stay and then separate from them. It was acknowledged that being around other individuals affected by trauma could make it more difficult to regulate one’s own emotions:

It’s not easy being in here either because there is so much, well everybody’s here for trauma…So again, you have got all of these nervous systems walking around that cannot regulate, well certainly not by themselves. And so, learning to regulate yourself is difficult.

#### 7d: importance of continuity of care and service integration

Participants asserted the importance of continuity of care following an inpatient stay, noted as an area of difficulty for the clinic and other mental health services: “Just leaving and not being connected to anyone. I found that really hard…[Same with] public systems…I just felt like there was no bridge between getting out and functioning on the outside.” Support with basic needs after leaving the clinic, such as accommodation and finances, was seen as necessary to sustain positive changes, as explained by one participant:

You really do want to see… sustainable, kind of change, changes that can be created through the programs. But if someone’s leaving there, and their primary issue is housing, their brain their kind of stress levels are going to be elevated and they are not necessarily going to get the benefit of that respite or when they come out, they are not going to be better off than when they came in.

Participants recommended employing a practitioner to support with navigating services and addressing other psychosocial needs, such as a social worker or advocate. Some were concerned about the ability to translate changes to the outside world and maintain them, emphasizing a desire for ongoing therapeutic support after the program. Those who engaged with the weekly day program saw this as beneficial for providing continuity of care:

Because sometimes when we go home after having an experience here… you have had people talking to you and supporting you all the time. And then at home you have not got that…And so coming up once a week is really good because it reaffirms everything.

#### 7e: other recommendations for improvement

Participants provided additional recommendations to improve the service to meet the needs of specific groups, accessibility and availability. Some discussed the importance of providing a neuro-affirming space for neurodivergent victim-survivors and staff training in working with neurodivergence. It was also recommended that services be more sensitive to the needs of individuals affected by disordered eating, with some stating that the service had sometimes been triggering for eating disorder symptoms. A recommendation to provide more spiritual support was also made. Furthermore, participants expressed a desire for more comfortable communal spaces to socialize with other women at the service, and more regular psychiatrist and psychologist appointments. Furthermore, it was noted that administrative difficulties created barriers and could lead already apprehensive individuals to avoid seeking help, therefore, improvements in administrative access to the service were recommended.

## Discussion

This study investigated the impact of Australia’s first women’s-only, holistic, trauma-and-violence-informed recovery service through developing an understanding of the experiences of program participants, and explored victim-survivors’ service systems experiences, recovery journey, and preferences for support. Thematic analysis resulted in seven themes that highlighted the benefits of the holistic support program for mental, physical, and social health, supporting the efficacy of such frameworks in promoting recovery and healing. Victim-survivors outlined existing barriers in current service systems and favored the implementation of trauma-informed, empowerment-based, relational, and holistic approaches to recovery support.

### Experiences prior to service entry

The impacts of DFSV that participants experienced were pervasive, complex, and widespread across multiple domains of health and functioning (3b). The effects relating to affect regulation, self-perception, shame, and self-blame aligned with findings from previous research into the unique psychological harm of DFSV (Herman, 2004; Anderson et al., 2012; Carman et al., 2023). As a result, trauma-focused therapy was identified as inherently challenging, requiring intensive emotional and cognitive work facilitating the rewiring of deeply entrenched neural pathways. Recovery was described as a multidimensional, non-linear, and lifelong process of progress and healing, involving integrating of learning over time, becoming “unstuck,” and a combination of both progress and setbacks (3a). These findings support scholars’ previous conceptualizations of recovery, contending its multidimensional and non-linear nature (Carman et al., 2023; D’Amore et al., 2021; Flasch, 2020), extending this by posing recovery as a challenging process of learning, transformation, and freedom from stagnation. This aligns with the idea of post-traumatic growth, with growth referenced by victim-survivors at various points; prior research demonstrates that many victim-survivors report post-traumatic growth across various domains, including an appreciation of their strengths and developing new perspectives (Elderton et al., 2017). The participants’ experiences additionally suggest that recovery does not necessarily indicate an absence of symptoms but the capacity for growth and resilience despite the experience of ongoing struggle, as discussed in Flasch (2020). The varied conceptualizations further demonstrate the unique nature of the recovery journey for each individual. Services that support victim-survivors need to take into account the challenges, complexities, and resilience inherent in recovery from DFSV, and the provision of long-term, ongoing, and individualized recovery support is necessary.

Participants’ previous experiences with mental health services were often harmful and disempowering, and trauma was often overlooked, dismissed, or invalidated (1a). This aligns with extensive previous research across Western, high-income countries indicating a high prevalence of negative and harmful service experiences for victim-survivors of DFSV (Kennedy et al., 2023; Murvartian et al., 2023; Ravi et al., 2022; Taylor, 2020). Participants in the current study experienced an overreliance on medication in clinical settings that negatively impacted their functioning and reduced their sense of autonomy. Previous research indicates that women exposed to DFSV use psychotropic medication at higher rates than the general population, independent of their level of mental distress (Stene et al., 2010). Taken together, these findings suggest that medications may be prescribed at higher rates for woman affected by DFSV regardless of their necessity, utility, or of client preference. To prevent a replication of the dynamics of disempowerment and control that occurred during previous experiences of abuse (Oram et al., 2022; Pemberton and Loeb, 2020), empowerment-based practices that promote survivor autonomy are necessary in clinical settings; for example, careful consideration of participant preferences and the benefits or drawbacks of treatment approaches, and collaboratively offering treatment options.

The systemic barriers and limitations to accessibility discussed by participants included costly insurance premiums, long waitlists, and limited practitioner availability (1b). These conditions reflect prior research in the Australian context (De Boer et al., 2022), and findings that the efficacy of Australia’s mental health system is constrained by underfunding (National Mental Health Commission, 2024; Salter et al., 2020). Participants discussed how these barriers disproportionately impact marginalized individuals, highlighting the intersection between trauma and social disadvantage that further impedes access to supports. Policy reform must not only mandate adequate funding for DFSV services but also address the broader inequities that perpetuate negative outcomes, such as by implementing poverty-reduction strategies, targeting discrimination, and increasing employment opportunities. Mental health systems have been shown to be inequitable (Dawadi et al., 2024), and services such as the RCT are extremely limited. Thus, participants advocated for such programs to be publicly funded to address an unmet, critical systemic need.

Participants valued a trauma-informed approach to support and advocated for more trauma-informed mental health services (2a), indicating that implementation of TIC in the current system remains inadequate and requires significant efforts to meet the required standard. This supports previous research suggesting that TIC’s application, in the Australian context, lacks consistency and cohesion (Quadara and Hunter, 2016). Participants additionally emphasized the need for more training and education for practitioners. Previous research indicates that mental health professionals feel underprepared and inadequately trained in relation to supporting DFSV victim-survivors, and that formal training increased skill and confidence (Sutton et al., 2020), while WHO guidelines advocate for specialized training in DFSV for primary care practitioners (World Health Organization, 2013). Furthermore, the current findings demonstrate that gender-specific, trauma-focused settings are crucial for women and AFAB individuals affected by DFSV, as this was noted as a primary benefit of the service (2b). This is particularly important given their disproportionate exposure to violence perpetrated by men (World Health Organization, 2024). These settings provide the safety necessary for healing, while encouraging help-seeking and engagement. All service reform must uphold the expertise of victim-survivor knowledge, consulting victim-survivors at all levels of policy design and implementation to ensure service systems adequately and effectively promote healing.

### Experiences of the s-CAPE program

The overwhelming majority of participants reported that the s-CAPE program was beneficial for their recovery after DFSV, reporting beneficial changes and improvements across a range of health and wellbeing domains, including mental, physical, and social health, trauma, coping skills, cognition, self-compassion, medication usage, and intrinsic motivation (Theme 5). The findings are supported by previous research indicating that high intensity and multi-component interventions improved mental health outcomes in DV victim-survivors (Micklitz et al., 2024). The program was described by some participants as “life-changing” and “what’s made the difference.” It was evidently beneficial to their quality of life and wellbeing and supported them to engage in meaningful activities. The changes considered most beneficial by participants included developing awareness and understanding of their trauma–which facilitated connecting with emotions, reduced feelings of shame, allowed for recognition and intervention of symptoms, and fostered empowerment and self-compassion–improvements in physical health, changes in medication, and increased social connection. All participants reported sustained improvements in physical health, which many attributed to the trauma-informed nature of the exercise program, highlighting the efficacy of implementing trauma-informed practices across all areas of holistic supports. Previous research demonstrates the benefits of trauma-informed approaches to health outcomes (Chu et al., 2024; Liu et al., 2024). Changes in medication surprisingly arose as a significant benefit for many participants that improved cognitive and physical functioning and increased connection with emotions, providing further evidence for the need to consider the impacts of medication and offer alternative treatment options where viable.

Essential components of the s-CAPE program that emerged from participant descriptions included empowerment and relational-based approaches–characterized by respect, autonomy, and empathy–centering safety, group work, and holistic supports. Participants highlighted the cruciality of feeling supported and understood and being treated with authentic compassion when receiving recovery support, which for some was the most valued aspect of their experience with the program (4b). This reflects previous findings across 20 countries suggesting that victim-survivors who felt unconditional support and empathy from service providers were more likely to access and continue engagement with various services (Ravi et al., 2022). Participants additionally appreciated the client-led nature of the program, including opportunities to exercise choice, voice, and control (4a), highlighting the importance of empowerment-based models of care and affirming these principles as fundamental to TIC and TVIC (Substance Abuse and Mental Health Services Administration, 2017; Tarzia et al., 2020; Wathen and Mantler, 2022). DV literature suggests that when victim-survivors experience greater control, voice, and empowerment when engaging with services, they report increased satisfaction, help-seeking, post-traumatic growth, and improved mental health outcomes (Cattaneo and Goodman, 2015; Elderton et al., 2017). Training protocols should thus prioritize the development of relational skills and DFSV services should develop programs that emphasize opportunities for agency and voice.

Additionally, participants reported feeling safer at the RCT than in previous supports, which facilitated recovery processes and produced psychological benefits. Support at the RCT allowed victim-survivors to foster emotional safety, which for some was an unfamiliar concept, and its development resulted in increased physical safety (4c). These findings highlight the importance of providing an emotionally and physically safe environment, a key component of TVIC (Wathen and Mantler, 2022), and establishing safety as a prerequisite for further recovery processes. Furthermore, the holistic nature of the s-CAPE program was highly valued by participants (4e); participants reported that the holistic supports facilitated more positive outcomes across health and wellbeing dimensions, aligning with previous literature demonstrating the benefits of holistic support for victim-survivors of sexual violence (Hegarty et al., 2017) and upholding recommendations from the WHO guidelines (World Health Organization, 2013). Holistic approaches allow for the comprehensive promotion of recovery across various domains.

The social support and improvements in social functioning resulting from engagement with the RCT were considered a central and transformative benefit of the program for all participants. Social connections established through the program were a source of emotional wellbeing, values learning, empowerment, relief, and shared understanding (6a). Further, group work was identified as one of the most beneficial components of the program, likely underpinned by these benefits. Peer support is a key principle of TIC (Substance Abuse and Mental Health Services Administration, 2017) and prior research emphasizes the benefits of group interventions in fostering social support, sharing of knowledge and validation (Micklitz et al., 2024). Many participants who had previously withdrawn socially reported increased motivation to re-engage with social connections outside of the clinic as a result of the program. This finding is particularly important as DFSV involves disconnection and disempowerment, and social support is considered one of the most impactful factors in supporting recovery (Alcantud et al., 2021; Blasco-Ros et al., 2010; Van der Kolk, 2015). Furthermore, participants reported that support to develop healthy social connections resulted in improved relationships with others, recognizing the negative impacts of problematic relationships, thus improving safety, and improved ability to advocate for themselves (6b). Therefore, social support and connection facilitate progress in both the interpersonal and intrapersonal components of recovery (Flasch, 2020) and services should thus establish environments that promote the development of safe, supportive relationships, social learning, and opportunities for peer support.

### Suggested service improvements

Participants identified a number of challenges and areas for improvement (Theme 7). Safety was impacted by factors such as inconsistent experiences with staff, high turnover, shortages leading to reliance on unfamiliar staff, and medication mismanagement. The significant challenges related to staffing is concordant with the systemic shortage of mental health staff across Australia’s mental health system (National Mental Health Commission, 2024) and highlights the need for sufficient funding and investment in education and training. The fact that participants reported aspects of the program that were less trauma-informed illuminates the difficulty of shifting entrenched practices in the mental health system, and the need for continued and purposeful effort, collaboration, and organization to ensure widespread and efficacious implementation of trauma-informed practice. Continuity of care was regarded as critical for recovery and stability post-discharge and considered lacking. This is particularly important as homelessness is a significant risk for DFSV victim-survivors (Baker et al., 2010), highlighting the need for coordinated care follow-up, such as by a dedicated practitioner. Stress relating to finances and accommodation hindered efficacy of treatment. Therefore, integrated service models are recommended to promote sustained safety and wellbeing throughout the recovery journey and mitigate the risk of unmet basic needs. Recommendations also highlighted barriers for specific groups, such as neurodivergent individuals. Given that autistic women are more likely to experience violence (Cazalis et al., 2022), more specialized services are needed, and further research into recovery frameworks for neurodivergent individuals would be beneficial.

### Limitations

This study comprehensively explored the impact of a novel framework for supporting recovery after DFSV, and provided further insight into victim-survivor experiences engaging with mental health services, conceptualizations of recovery, and recommendations for service improvement from the victim-survivor perspective. This study is not without its limitations. Study participants had been engaged with the service for different lengths of time and had experienced various levels of support; therefore, results may not reflect entirely consistent experiences. However, all participants had completed at least 2 weeks of Phase One and the findings broadly reflect a comprehensive range of experiences with the service. As the RCT is a privately funded service and participants were largely of Anglo-Australian or British background, the current findings represent the experiences of a small and selected socioeconomic and cultural subset of victim-survivors. Given the intersection of violence with other experiences of oppression (Crenshaw, 1991) and the increased risk of women from multicultural backgrounds experiencing violence (Rees et al., 2022), these findings may not be generalizable to a large proportion of individuals affected by DFSV and those not availing support in a clinical setting.

## Conclusion

This study employed a qualitative methodology to understand the impact of a holistic, trauma-and-violence-informed framework on the recovery of victim-survivors of DFSV, implemented at Australia’s first women’s-only, trauma-specific hospital. Participant perspectives indicated that the program was largely beneficial for victim-survivor recovery across the domains of mental, physical, and social health, although some aspects of the program required further development. This study provides a comprehensive exploration of the impact of a novel framework for recovery from trauma after DFSV across different domains, while offering further insights into the experiences of recovery of women affected by DFSV. Furthermore, the current findings deepen understanding of the experiences of woman affected by DFSV who engage with service systems and their recommendations for service improvements. Findings indicate the crucial need for more gender-specific, trauma-focused services, and for continual effort, collaboration, and investment in developing integrated trauma-and-violence-informed services to mitigate harm and promote recovery and wellbeing. This will lead to more sustainable, effective recovery and healing for victim-survivors of domestic, family and sexual violence. Further research should explore the longitudinal impacts of such programs and involve more socioeconomically and culturally diverse populations.

## Data Availability

The datasets presented in this article are not readily available because due to the sensitive nature of the qualitative data and the potential risk of identifying participants who have experienced domestic, family, and sexual violence, the datasets generated and analyzed during the current study are not publicly available. Data sharing would compromise participant confidentiality and safety. Limited, de-identified excerpts may be available upon reasonable request and with appropriate ethical approval. Requests to access the datasets should be directed to Lata Satyen, lata@deakin.edu.au.
